# Proteomic alterations in outer membrane vesicles of carbapenem-resistant *Klebsiella pneumoniae* isolated from sepsis patients

**DOI:** 10.1515/med-2026-1376

**Published:** 2026-02-24

**Authors:** JingJing Kang, LinLin Xu, TengFei Xiao

**Affiliations:** Department of Clinical Laboratory, Yancheng First Hospital, Affiliated Hospital of Nanjing University Medical School, The First People’s Hospital of Yancheng, Yancheng City, Jiangsu Province, China; Department of Clinical Laboratory, Yancheng Third People’s Hospital, Affiliated Hospital 6 of Nantong University, Yancheng City, Jiangsu Province, China; Department of Clinical Laboratory, Yancheng Third People’s Hospital, the Affiliated Hospital of Jiangsu Vocational College of Medicine, Yancheng City, Jiangsu Province, China

**Keywords:** carbapenem-resistant *Klebsiella pneumoniae*, outer membrane vesicles, sepsis, antibiotic resistance, differential protein analysis

## Abstract

**Objectives:**

Carbapenem-resistant *Klebsiella pneumoniae* (CRKP) is a major clinical and public health threat due to its high mortality in sepsis and the complexity of its resistance mechanisms, which greatly limit therapeutic effectiveness. Outer membrane vesicles (OMVs) secreted by Gram-negative bacteria harbor various virulence factors and resistance determinants, and may facilitate long-distance pathogenic communication. However, the OMV protein composition of CRKP isolated from sepsis patients remains poorly understood. This study aimed to characterize the protein cargo of OMVs derived from clinical CRKP isolates and to identify differential proteins and pathways associated with bacterial pathogenicity and carbapenem resistance.

**Methods:**

Three CRKP and three carbapenem-sensitive *K. pneumoniae* (CSKP) were isolated from blood cultures of patients with sepsis, and then their OMVs were extracted. Transmission electron microscopy (TEM), nanoparticle tracking analysis (NTA), and SDS-PAGE were used for characterization. Quantitative proteomic profiling was performed using LC–MS/MS, followed by differential expression analysis, Gene Ontology (GO), KEGG pathway enrichment, and protein–protein interaction (PPI) network analysis.

**Results:**

A total of 1,193 OMV proteins were identified, with CRKP-OMVs containing substantially more unique proteins (140 vs. 5 in CSKP-OMVs) and significantly increased overall protein abundance. Among the 199 differential proteins, 180 were upregulated in CRKP-OMVs. Most differential proteins were localized to the membrane or cytoplasm, and were enriched in enzymatic functions and pathways including β-alanine metabolism, O-antigen nucleotide sugar biosynthesis, Lipopolysaccharide biosynthesis, and β-lactam resistance. Key proteins such as adhE, NDK, treA, and ackA were markedly elevated, while galE and Ter family proteins were uniquely present in CRKP-OMVs, indicating potential roles in resistance and pathogenicity.

**Conclusions:**

CRKP-derived OMVs from sepsis patients exhibit distinct protein enrichment patterns associated with membrane functions, metabolic remodeling, and antibiotic resistance. These findings provide insights into CRKP pathogenesis and highlight candidate OMV-associated proteins that may serve as targets for antimicrobial strategies or vaccine development.

## Introduction

*Klebsiella pneumoniae*, a facultative anaerobic bacterium, is the second most commonly isolated Gram-negative pathogen in both community-acquired and nosocomial infections [1], [2], [3]. It primarily arises as a secondary infection following surgical procedures involving the urinary and digestive systems, as well as pulmonary infections, which can lead to bloodstream infections upon failure of immune resistance [4]. The prognosis for patients with *K. pneumoniae* sepsis is notably poor, with a high mortality rate persisting within 30 days post-infection [5]. Research indicates that *K. pneumoniae* strains isolated from septic patients generally exhibit sensitivity in drug susceptibility tests; clinical applications of cephalosporins and carbapenems have demonstrated favorable antibacterial efficacy. However, it is concerning that the rate of drug resistance among *K. pneumoniae* strains associated with bloodstream infections has escalated significantly in recent years [6]. Carbapenem-resistant *K. pneumoniae* (CRKP) has emerged as a critical issue for public health safety, often resulting in prolonged hospital stays and increased mortality rates [7]. The mechanisms underlying CRKP’s resistance are multifaceted; factors contributing to extensively drug-resistant (XDR) or pandrug-resistant (PDR) phenotypes include the production of carbapenemases, deficiencies in outer membrane proteins, and overexpression of efflux pumps [7], 8]. In addition to precise antimicrobial therapy, such as correlating drug resistance genes with minimum inhibitory concentration (MIC) results, there is an urgent need for new targeted therapies and antimicrobial strategies to enhance research and development efforts against this formidable pathogen.

Outer membrane vesicles (OMVs) are nanoscale, spherical entities that contain a variety of virulence factors and drug resistance genes secreted by Gram-negative bacilli during the processes of adhesion and invasion [9]. OMVs offer several advantages in initiating infection. They originate from the extracellular membrane, serving as decoys to absorb antibiotic exposure from bacteria, while also releasing vesicular enzymes such as β-lactamases that can directly degrade antibacterial agents [10]. The long-distance communication capabilities of OMVs render bacterial-host interactions less reliant on live bacteria themselves [11]. Research has demonstrated that OMVs can facilitate horizontal transfer of drug-resistant plasmids and even penetrate the blood-brain barrier, exhibiting a remarkable organ-specific enrichment function [12], 13]. Investigations into *K. pneumoniae*-derived OMVs have progressively advanced; some studies have confirmed that these vesicles elicit a robust inflammatory response when co-cultured with human bronchial epithelial cells (BEAS-2B) [14]. In cases of sepsis, OMVs play a pivotal role in undermining traditional anti-infective treatments triggered by CRKP [15]. These vesicles carry substantial amounts of Lipopolysaccharides (LPS) and lipoproteins, acting as more effective activators than purified LPS alone by activating the TLR4 pathway and instigating systemic inflammatory response syndrome [16].

Due to the unique characteristics of OMVs, they offer novel insights into elucidating the mechanisms underlying CRKP resistance and in developing new rapid diagnostic reagents. The outer membrane proteins and pathogen-related molecules present in OMVs also facilitate their potential use as vaccines for the prevention of CRKP infections [17]. Researchers have engineered bovine serum albumin (BSA) nanoparticles to ensure a uniform and stable distribution of OMVs, thereby enhancing the internal structure of OMV-based vaccines developed against CRKP, which has led to a significant improvement in immune efficacy [18].

The role of OMVs and their protein components secreted by CRKP has become increasingly elucidated with advancements in proteomics technology. Existing literature confirms that during imipenem treatment, there is an increase in the formation of OMVs, enhanced cell lysis, and a significant release of extracellular OXA-58 [19], 20]. However, the alterations in protein components and functions of *K. pneumoniae*-derived OMVs during carbapenem exposure in the context of sepsis remain poorly understood. The objective of this study is to analyze the protein components of OMVs secreted by *K. pneumoniae* isolated from patients with sepsis, thereby providing novel theoretical support for the development of drugs and vaccines targeting OMVs to combat CRKP invasion.

## Materials and methods

### Bacterial strain and measurements of MICs

*K. pneumoniae* strains isolated from blood cultures of sepsis patients at The First people’s Hospital of Yancheng in 2024 were collected, with three CRKP and three CSKP strains identified among them. The inclusion criteria for sepsis included bacteriological evidence, identifiable infection foci, and a Sequential Organ Failure Assessment (SOFA) score of ≥2 [21]. The purified strains were subsequently transferred into strain preservation tubes and stored at −80 °C.

*K. pneumoniae* species identification was conducted using the Autof ms1000 (Autobio, China), while minimum inhibitory concentrations (MICs) were determined with the VITEK-Ⅱ system (Biomerieux, France). *Escherichia coli* ATCC 25922 was employed as a quality control reference for both experiments. The MIC results were categorized into susceptible, intermediate, and resistant (S/I/R) based on the breakpoints established in the Clinical and Laboratory Standards Institute (CLSI) guidelines from the previous year. In this study, CRKP strains exhibited resistance to imipenem, doripenem, and meropenem. Conversely, CSKP strains displayed a normal non-mutated genotype and demonstrated sensitivity to all drugs listed in Table 1.

**Table 1:** MICs results of *Klebsiella pneumoniae* strains.

	CRKP1	CRKP2	CRKP3		CSKP1	CSKP2	CSKP3	
Polymyxin	≤0.5	≤0.5	≤0.5	S	0.5	1	0.5	S
Tigacycline	1	0.5	0.5	S	0.5	1	0.5	S
Compound sulfamethoxazole	≥320	≥320	≥320	R	≤20	≤20	≤20	S
Norfloxacin	≥16	≥16	≥16	R	≤0.5	≤0.5	≤0.5	S
Moxifloxacin	≥8	≥8	≥8	R	≤0.25	≤0.25	≤0.25	S
Levofloxacin	≥8	≥8	≥8	R	≤0.12	≤0.12	≤0.12	S
Ciprofloxacin	≥4	≥4	≥4	R	≤0.25	≤0.25	≤0.25	S
Nalixic acid	≥32	≥32	≥32	R	≤2	≤2	≤2	S
Doxycycline	8	8	8	R	1	1	1	S
Minocycline	≥16	≥16	≥16	R	2	2	2	S
tetracycline	8	8	8	R	≤1	≤1	≤1	S
amikacin	≥64	≥64	≥64	R	≤2	≤2	≤2	S
tobramycin	≥16	≥16	≥16	R	≤1	≤1	≤1	S
Meropenem	≥16	≥16	≥16	R	≤0.25	≤0.25	≤0.25	S
Imipenem	≥16	≥16	≥16	R	≤0.25	≤0.25	≤0.25	S
Donipenem	≥8	≥8	≥8	R	≤0.12	≤0.12	≤0.12	S
aztreonam	≥64	≥64	≥64	R	≤1	≤1	≤1	S
cefotetan	≥64	≥64	≥64	R	≤4	≤4	≤4	S
Cefoperazone/Sulbactam	≥64	≥64	≥64	R	≤8	≤8	≤8	S
cefepime	≥32	≥32	≥32	R	≤0.12	≤0.12	≤0.12	S
cefazoxime	≥64	≥64	≥64	R	≤1	≤1	≤1	S
ceftazidime	≥64	≥64	≥64	R	0.25	0.25	0.25	S
cefotaxime	≥64	≥64	≥64	R	≤1	≤1	≤1	S
cefpoxime	≥8	≥8	≥8	R	≤0.25	≤0.25	≤0.25	S
Cefuroxime axetil	≥64	≥64	≥64	R	2	2	2	S
cefuroxime	≥64	≥64	≥64	R	2	2	2	S
cephalothiophene	≥64	≥64	≥64	R	≤2	≤2	≤2	S
Piperacillin/Tazobactam	≥128	≥128	≥128	R	≤4	≤4	≤4	S
Amoxicillin/clavulanic acid	≥32	≥32	≥32	R	4	4	4	S

### Isolation and purification of OMVs

A single colony in the logarithmic growth phase was inoculated into lysogeny broth (LB) to prepare a bacterial suspension, which was shaken evenly at 37 °C and 180 rpm for 24 h until the absorbance at 600 nm (OD_600_) reached approximately 1. Subsequently, the harvested bacterial suspension was centrifuged at 12,000 g for 30 min at 4 °C to remove intact bacteria and large debris. The resulting supernatant was carefully collected to avoid disturbing the pellet and subsequently passed through a 0.22 µm sterile filter to eliminate residual cells or apoptotic bodies. The filtrate was transferred to ultracentrifuge tubes and subjected to ultracentrifugation at 100,000 g for 70 min at 4 °C (Beckman Coulter, USA).

The OMV-containing pellet was gently resuspended in 100 µL of PBS and filtered again through a 0.22 µm membrane to further remove protein aggregates. Throughout the isolation procedure, particular care was taken to minimize contamination from cytoplasmic or periplasmic components. As part of routine quality control, the post-ultracentrifugation supernatant was visually inspected and did not contain vesicle-like structures, indicating efficient pelleting and specificity of OMV separation. Additionally, sterility of the final OMV preparations was confirmed by plating the filtrate onto LB agar and incubating for 48 h, during which no bacterial colonies were observed.

The high purity of the OMV fraction was further supported by subsequent proteomic profiling, which did not show enrichment of typical cytoplasmic, periplasmic, or inner-membrane marker proteins, whereas outer-membrane–associated proteins predominated.

### Transmission electron microscopy (TEM)

Electron microscopy imaging of OMVs was conducted using TEM (Thermo Scientific, USA). A volume of 10 μL of the OMV solution was carefully aspirated onto a copper mesh that had been wrapped with a sealing membrane and allowed to stand for 10 min. Subsequently, excess liquid was removed using filter paper. The OMVs were then treated with 10 μL of 2 % uranyl acetate for a duration of 2 min, followed by another blotting step with filter paper. The grids were air-dried for approximately 10 min efore imaging. Electron micrographs were acquired at an accelerating voltage of 100 kV using a TEM system (Thermo Scientific, USA). As part of routine quality control during OMV preparation, the post-ultracentrifugation supernatant was visually inspected and did not contain vesicle-like structures, consistent with the successful pelleting and specificity of OMV isolation.

### Nanoparticle tracking analysis (NTA)

The particle size distribution and concentration detection of OMVs in this study were conducted using a NTA system (Particle Metrix, Germany). To calibrate the instrument, a polystyrene microsphere standard (100 nm, 1:250,000) was introduced into a sample cell that had been decontaminated with deionized water. The diluted OMVs in 1 × PBS were then transferred to the calibrated sample pool, which had also been washed with 1 × PBS, and subsequently irradiated with a laser beam. The light refracted by the OMVs following laser irradiation was captured using microscopy techniques. The particle size and concentration of the OMVs components were calculated based on a modified Stokes-Einstein equation.

### Protein extraction and protein concentration

The OMVs were combined with the sample lysate, phosphatase inhibitor, and protease inhibitor (Beyotime Biotechnology, China). The mixture was then placed on ice and subjected to ultrasonic disruption at 80 W for 2 min. Following this, the homogenate was centrifuged at 12,000 rpm for 10 min at 4 °C; the resulting precipitate was discarded and the supernatant was stored at −80 °C for subsequent protein concentration determination using a BCA kit (Thermo Scientific, USA). In accordance with the instructions provided by the BCA kit, 2 μL of protein solution was added to a 96-well plate. Subsequently, 200 μL of prepared chromogenic solution was added to each well and incubated at room temperature for half an hour. The absorbance value measured at 562 nm was then used to interpolate from a standard curve in order to calculate the protein concentration.

### Sodium dodecyl sulfate polyacrylamide gel electrophoresis (SDS-PAGE)

12 % SDS-PAGE was performed to separate the protein fractions in OMVs. After separation, the gel was cleaved and transferred to the eStain LG protein stain (genscript, China) for Coomassie brilliant blue staining.

### Protein digestion and mass spectrometry (MS) detection of OMVs

Dithiothreitol (DTT, 25 mM) was added to the OMVs solution, which was diluted to ensure uniform concentration and volume across all groups. The final concentration of DTT in each mixed solution was adjusted to 5 mM. Following a 55 °C incubation, different volumes of iodoacetamide were introduced into the samples after cooling them to room temperature, thereby increasing the DTT concentration to 10 mM. The samples were then protected from light for 20 min. To precipitate the proteins, precooled acetone – six times the volume of the reaction mixture – was added and incubated at −20 °C overnight. The resulting protein precipitate was dissolved in 100 μL of NH_4_HCO_3_ solution (50 mM) along with an enzymatic diluent (protein: enzyme ratio=50:1 by mass), followed by digestion at room temperature for 18 h. Subsequently, enzymatic hydrolysis was performed by adjusting the pH to 3 using phosphoric acid. The peptide fragments generated from this hydrolysis were desalted using SOLA™ SPE and subsequently dried under vacuum conditions. Finally, the product was re-dissolved and spiked with iRT peptide at a ratio of 1:10.

The proteomic data analysis in this study was commissioned to Shanghai OE Biotechnology Co., Ltd. (Shanghai, China). Adopting the mass spectrometer (Thermo, Bruker) paired with an Easyspray source (Thermo, USA) to analyze each group of OMVs. OMVs were fixed in a C18 column (15 cm × 75 µ m) and loaded into the EASY nLCTM 1,200 system (Thermo, USA) to capture MS/MS spectra ranging from 100 to 1,700 m/z. The flow rate was 300 nL/min and linear gradient was set as follow: 0–20 min, 5–22 % B; 20–24 min, 22–37 % B; 24–27 min, 37–80 % B; 27–30 min, 80 % B. Ion mobility is set from 0.7 to 1.3 Vs/cm2 and the collision energy range from 20 to 59 eV.

### Bioinformatics analysis of OMVs

Proteins with Unique Peptides ≥1 and detected in at least two replicates of each group were considered reliable for downstream analysis. The expression matrix was log_2_-transformed and used for quantitative comparison and functional annotation. Principal component analysis (PCA) was performed to evaluate overall differences between groups, and a relative standard deviation (RSD) box plot was used to assess the repeatability of samples within each group. Hierarchical clustering and heatmap visualization were applied to display protein expression patterns.

Functional annotation of proteins was conducted using Gene Ontology (GO; (http://www.geneontology.org/), Kyoto Encyclopedia of Genes and Genomes (KEGG; https://www.genome.jp/kegg/) and InterPro (https://www.ebi.ac.uk/interpro/). Protein–protein interaction (PPI) networks were constructed using STRING (https://string-db.org/), and the top 25 proteins ranked by connectivity were used to generate the interaction grid. Subcellular localization of proteins was obtained from the UniProt database (https://www.uniprot.org/).

Differentially expressed proteins between CRKP-OMVs and CSKP-OMVs were identified using a two-sample t-test with Benjamini–Hochberg false discovery rate (FDR) correction. Proteins with an absolute log_2_ fold change (log_2_FC) ≥1.5 and FDR<0.05 were defined as significantly differentially expressed.

## Results

### OMVs identification

**Figure 1:**
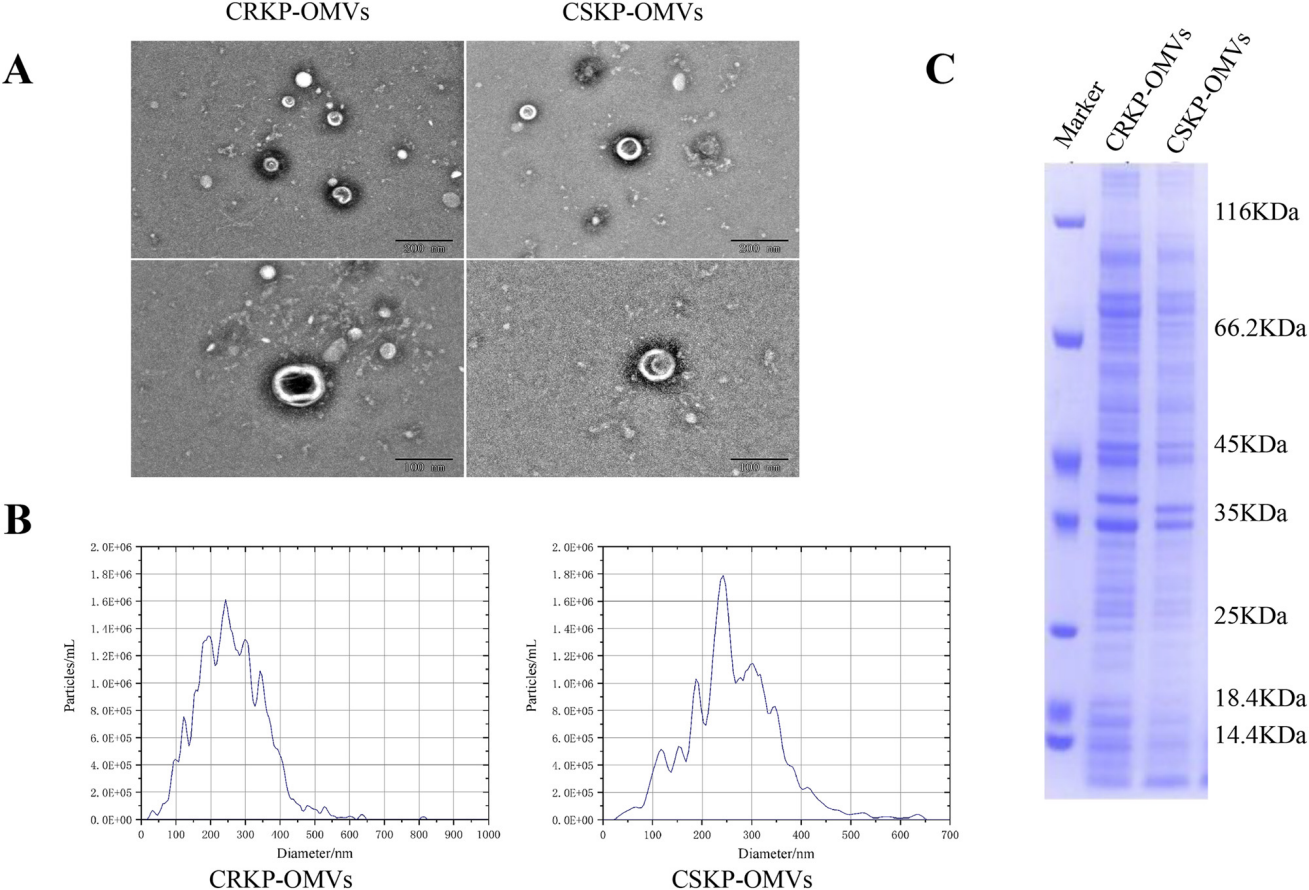
**OMVs identification.** (A) TEM images of the CRKP-OMVs and CSKP-OMVs. (B) NTA analysis of the CRKP-OMVs and CSKP-OMVs. (C) SDS-PAGE analysis of the CRKP-OMVs and CSKP-OMVs.

Three CRKP and three CSKP strains from blood culture of sepsis patients were grown in LB broth for 24 h, and then the supernatant was collected and purified for OMVs. The OMVs preparations showed no bacterial growth after plating on LB agar for 48 h, confirming the sterility and reliability of the extraction procedure. Measurement of protein concentration revealed that the total protein content of OMVs secreted by CRKP strains was 2.730 μg, 2.350 μg, and 2.470 μg, while that of OMVs secreted by CSKP strains was 2.640 μg, 2.690 μg, and 2.380 μg. No significant difference in OMV protein yield was observed between the strains. To verify the identity of OMVs, one each of CRKP-OMVs and CSKP-OMVs was selected to observe their morphology at the nanoscale using electron microscopy. As shown in Figure 1A, both preparations displayed typical spherical, bilayer-bound vesicles with similar size ranges and dispersed distribution patterns. NTA analysis further revealed that the average diameter of CRKP-OMVs was 249.5 nm, with a particle concentration of 3.4E+11 particles/mL. In comparison, the average diameter of CSKP-OMVs measured 242.5 nm, and their particle concentration was recorded at 4.6E+11 particles/mL (Figure 1B). SDS-PAGE profiling demonstrated that OMVs from both groups contained a broad spectrum of proteins with highly similar banding patterns, reflecting the overall consistency of OMV protein composition across isolates. Minor differences in band intensity – particularly around the ∼35 kDa region – were attributable to slight variations in loading amount rather than intrinsic biochemical differences (Figure 1C). Comprehensive analysis revealed no significant differences in the key characteristics of OMVs—including protein yield, morphology, particle size and distribution, and protein profile—isolated from the different strains in this study.

### Protein abundance analysis of CRKP-OMVs and CSKP-OMVs

**Figure 2:**
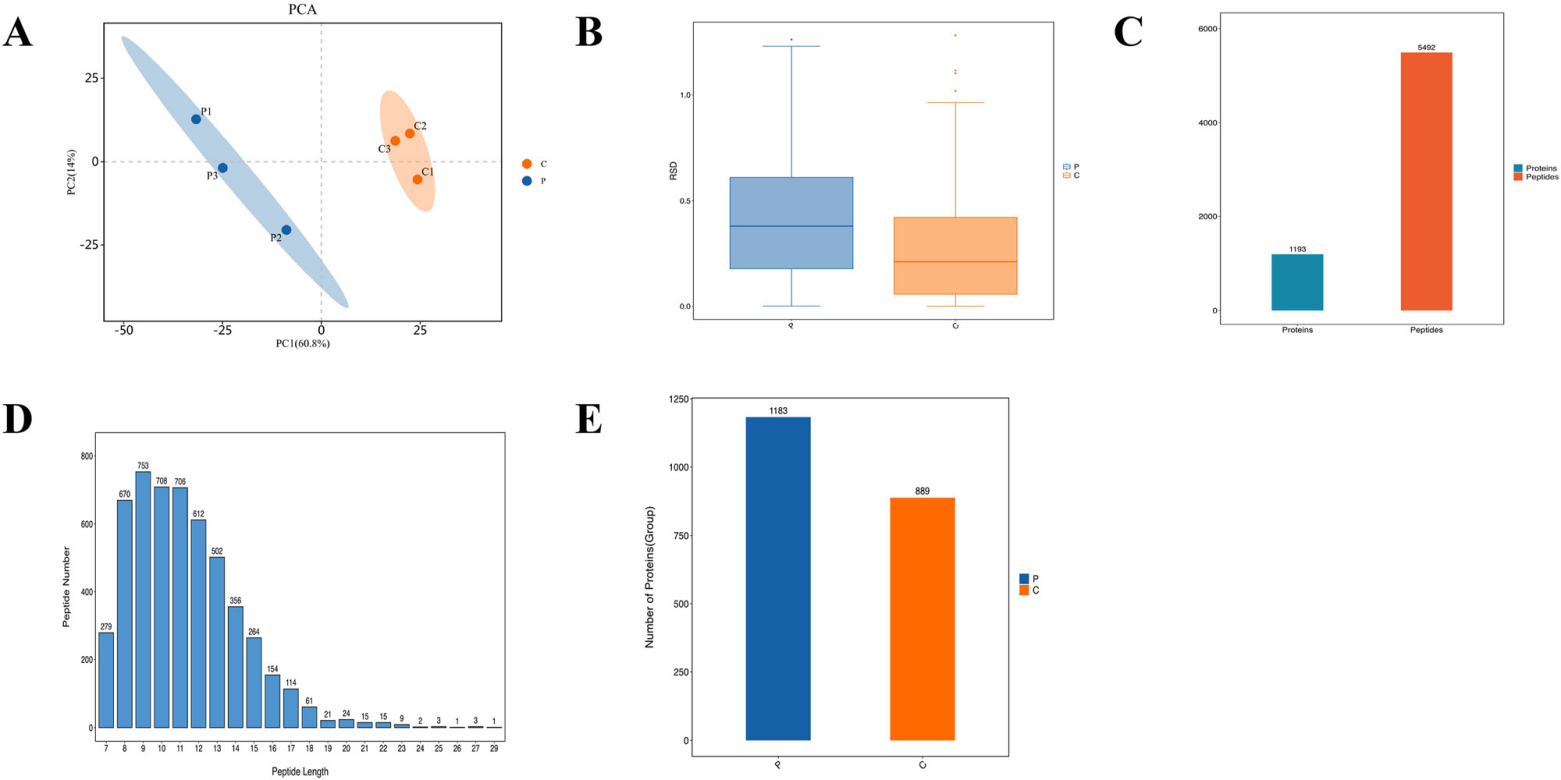
**Protein abundance analysis of CRKP-OMVs and CSKP-OMVs.** (A) PCA analysis of the expression of CRKP-OMVs and CSKP-OMVs credible proteins. (B) RSD box plot of CRKP-OMVs and CSKP-OMVs protein data. (C) Number of OMVs proteins, peptides detected by MS. (D) Peptide distribution of OMVs. (E) The number of detected proteins of CRKP-OMVs and CSKP-OMVs. P: CRKP-OMVs; C: CSKP-OMVs.

Protein abundance in CRKP-OMVs and CSKP-OMVs was determined by MS and data evaluation was performed to filtrate trustworthy proteins. PCA analysis proved that there were differences in the expression of believable proteins between CRKP-OMVs and CSKP-OMVs in different dimensions (Figure 2A), while PCA analysis and RSD box plot showed that the protein data within CRKP-OMVs and CSKP-OMVs groups were statistically consistent (Figure 2B). MS data tested that a total of 1,193 proteins and 5,492 peptides were detected (Figure 2C), and most of the peptides were distributed in 7–20 amino acids (Figure 2D). Interestingly, when 1,183 proteins can be accommodated in CRKP-OMVs but only 889 in CSKP-OMVs, the amount of protein increases significantly when *K. pneumoniae* changes from wild-type to carbapenem resistant (Figure 2E).

### Comparison of differential proteins between CRKP-OMVs and CSKP-OMVs

**Figure 3:**
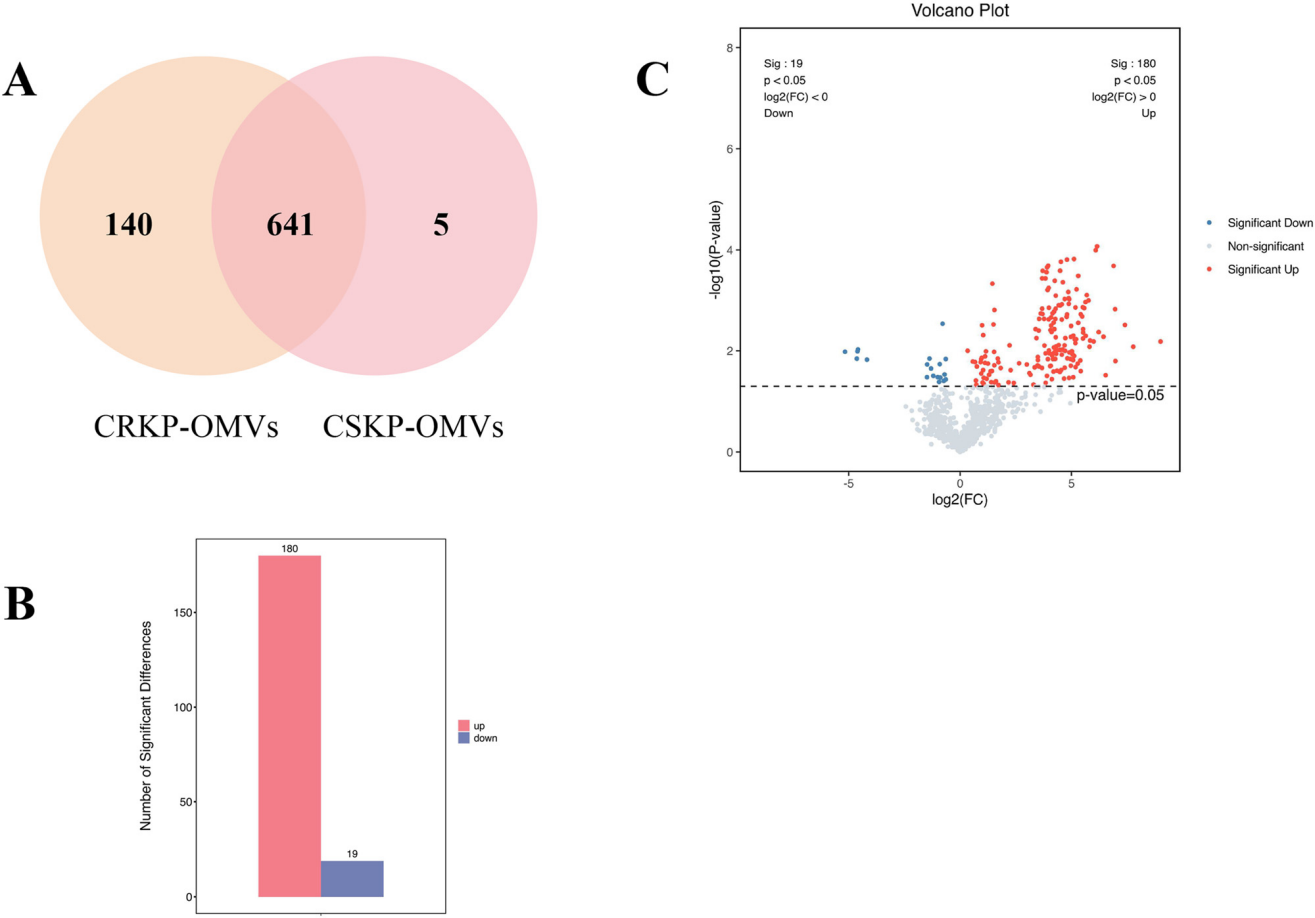
**Comparison of differential proteins between CRKP-OMVs and CSKP-OMVs.** (A) Venn diagram of differential proteins between CRKP-OMVs and CSKP-OMVs detected by mass spectrometry. (B) Compared with CSKP-OMVs, 180 proteins were up-regulated and 19 proteins were down-regulated in CRKP-OMVs. (C) Volcano plot of differential proteins between CRKP-OMVs and CSKP-OMVs. |log_2_FC| ≥1.5 and FDR <0.05. P: CRKP-OMVs; C: CSKP-OMVs.

Unique proteins and proteins with detection frequency of 3 are retained in the original data to better analyze and compare the differential proteins of CRKP-OMVs and CSKP-OMVs. According to the screened data, 641 proteins were shared by CRKP and CSKP derived OMVs, while 140 proteins were uniquely present in CRKP-OMVs and only 5 proteins were detected by CSKP-OMVs alone (Figure 3A). The proteome data were log transformed. CSKP-OMVs were set as the control group and CRKP-OMVs were set as the positive group. The differential proteins with log_2_FC (foldchange) ≥1.5 or ≤1/1.5 and p-value <0.05 obtained by t-test were retained. Differential expression analysis revealed a striking imbalance between the two groups: 180 proteins were up-regulated in CRKP-OMVs, whereas only 19 proteins were down-regulated (Figure 3B). As illustrated by the volcano plot, CRKP-OMVs displayed a markedly enriched set of up-regulated proteins (Figure 3C). These findings indicated that CRKP isolates released OMVs with a broader and more abundant proteomic cargo than CSKP isolates, suggesting substantial remodeling of OMV composition associated with carbapenem resistance.

### Functional analysis of differential proteins

**Figure 4:**
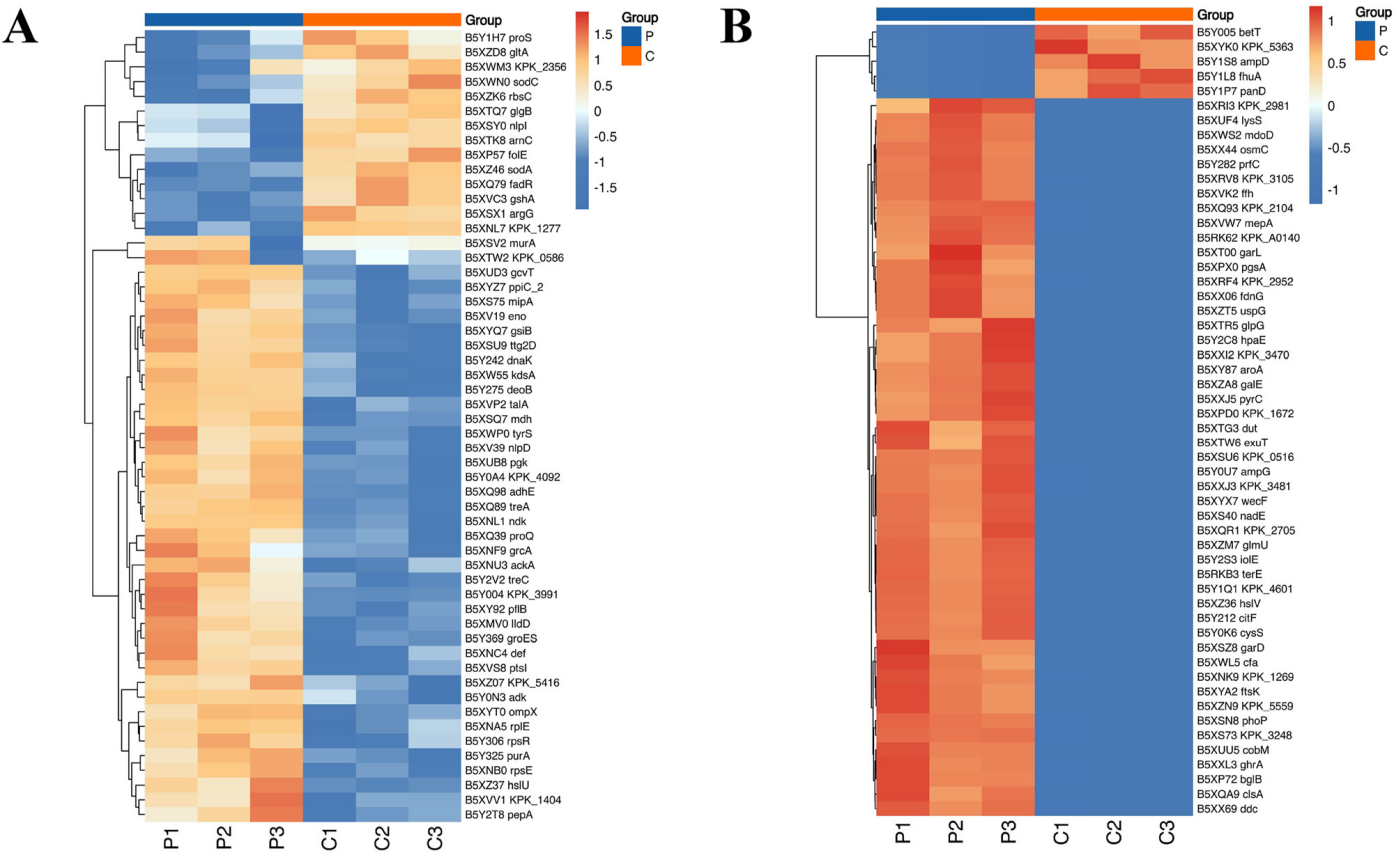
**Heatmap of differential proteins.** (A) Heatmap of 54 differential proteins shared by CRKP-OMVs and CSKP-OMVs. (B) Heatmap of the Top 50 unique proteins with increased expression and five unique proteins with decreased expression in CRKP-OMVs compared to CSKP-OMVs. |log_2_FC| ≥1.5 and FDR <0.05. P: CRKP-OMVs; C: CSKP-OMVs.

Cluster heatmaps were generated to visualize expression patterns of the differentially expressed proteins between CRKP-OMVs and CSKP-OMVs. Among the 54 proteins shared by the two groups, 14 proteins were downregulated and 50 proteins were upregulated (Figure 4A). In addition, unique proteins with elevated Top 50 expression and five unique proteins with decreased expression were selected (Figure 4B).

**Figure 5:**
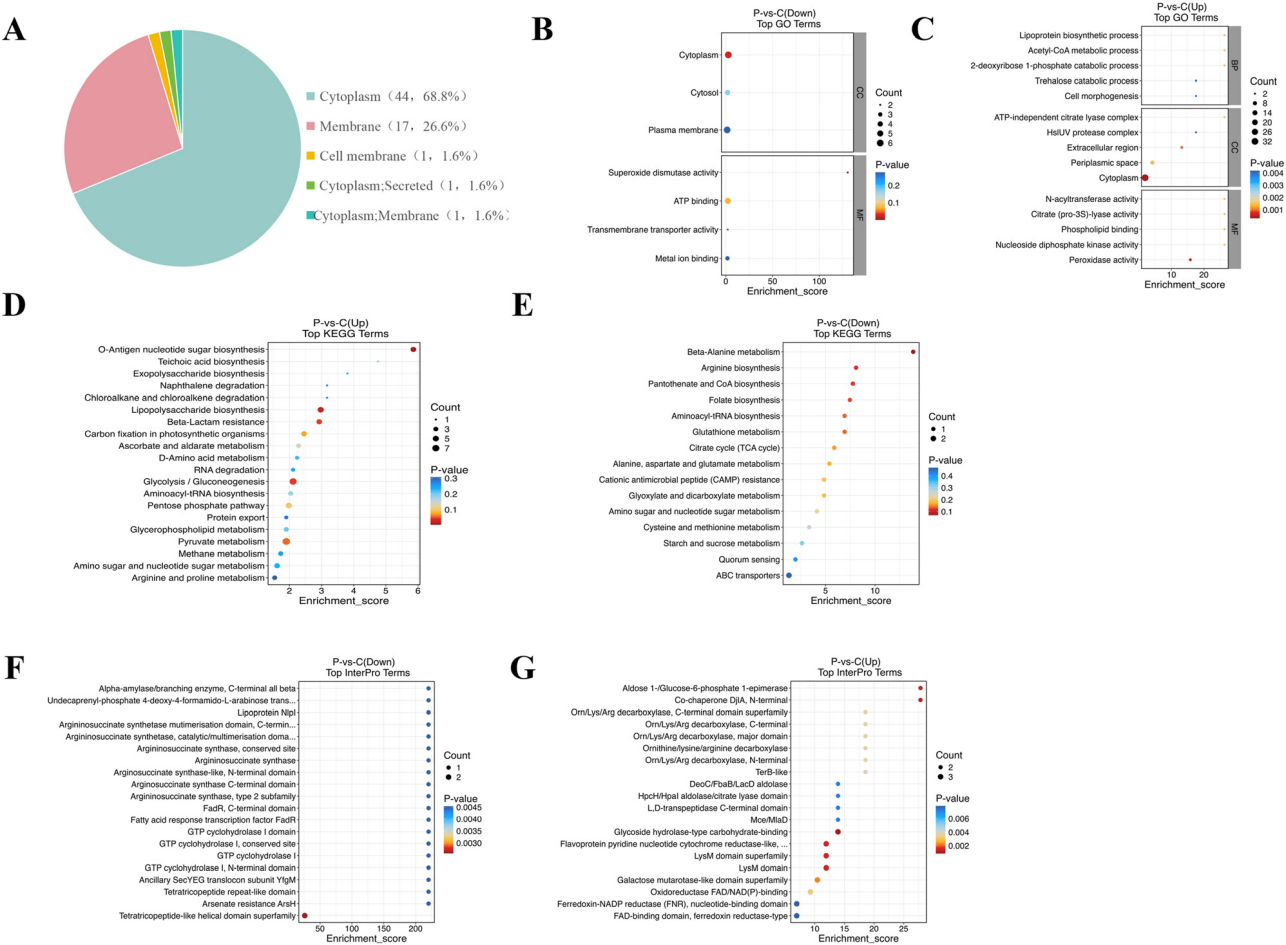
**Functional analysis of differential proteins between CRKP-OMVs and CSKP-OMVs.** (A) The subcellular localization of differential proteins in OMVs was determined by universal protein resources. (B–C) Go enrichment analysis was used to analyze the enrichment function and characteristics of differential proteins. (D–E) KEGG pathway database was used to describe the biological metabolic pathways of OMVs differential proteins. (F–G) The protein database interpro was used to comprehensively annotates the differential protein functions in OMVs. P: CRKP-OMVs; C: CSKP-OMVs.

To characterize the biological properties of the differential proteins, subcellular localization analysis indicated that most proteins were annotated as cytoplasmic (n=44), followed by membrane-associated proteins (n=17), with a smaller number distributed in periplasmic or secretory compartments (Figure 5A).

Go enrichment analysis showed that differential proteins were mainly associated with cytoplasmic components and functions related to metabolic processes, catalytic activity, and redox regulation (Fig. 5B and C). Down-regulated proteins were enriched in pathways associated with Superoxide dismutase activity, whereas up-regulated proteins showed enrichment in Peroxidase activity and periplasmic processes.

KEGG pathway analysis revealed that down-regulated proteins were significantly enriched in β-alanine metabolism, arginine biosynthesis, and pantothenate/coenzyme A (CoA) biosynthesis (Figure 5D). In contrast, up-regulated proteins were predominantly associated with pathways including O-antigen nucleotide sugar biosynthesis, Lipopolysaccharide biosynthesis, β-lactam resistance, glycolysis/gluconeogenesis, and pyruvate metabolism (Figure 5E).

Interpro domain enrichment further demonstrated that down-regulated proteins clustered within tetratricopeptide-like helical domain families, whereas up-regulated proteins were enriched in glycoside hydrolase–related carbohydrate-binding domains, aldose/glucose-6-phosphate epimerase domains, and chaperone-related N-terminal domains (Fig. 5F and G).

**Figure 6:**
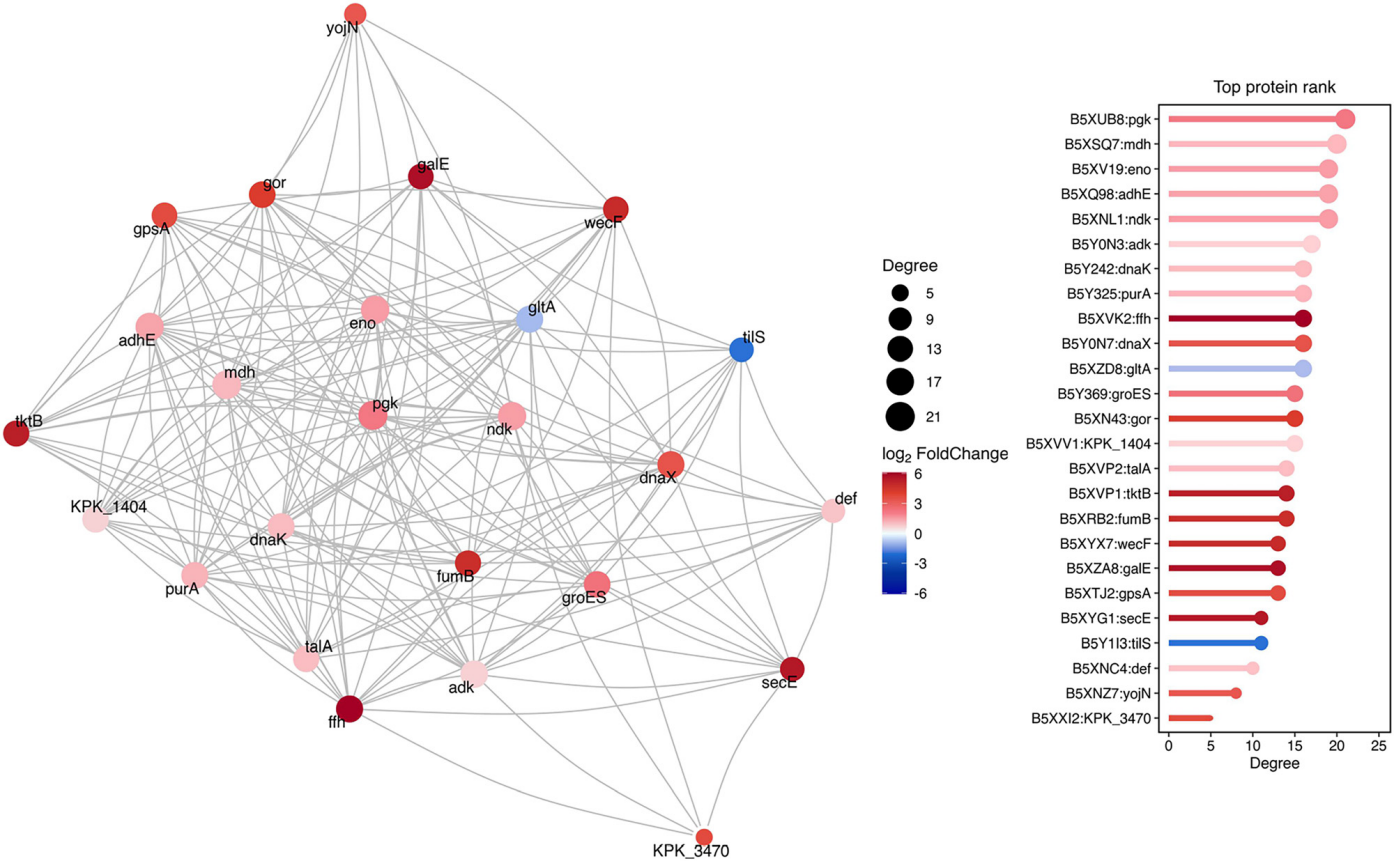
**Protein protein interaction (PPI) network analysis.** STRING database was used to predict and analyze the interactions between differentially expressed proteins in CRKP-OMVs and CSKP-OMVs.

PPI interaction analysis using STRING identified several proteins with high connectivity, including *pgk*, *mdh*, *eno*, *adhE*, *ndk*, *adk*, *dnaK*, and *purA*, highlighting key nodes within the differential OMV proteome (Figure 6).

## Discussion

Sepsis caused by *K. pneumoniae* has emerged as a significant concern for patients experiencing ineffective anti-infection treatments and poor prognoses following surgery, immunosuppressive therapy, and ICU admissions [22]. The increasing prevalence of CRKP strains isolated in clinical settings has led to a gradual decline in the efficacy of traditional β-lactam antibiotics, with some even becoming ineffective [2]. Carbapenems are commonly prescribed for CRKP infections in China; they primarily exert their antibacterial effects by inhibiting bacterial cell wall synthesis. However, the emergence of hydrolase production by bacteria, active drug efflux mechanisms, self-sealing processes that reduce drug uptake, and genetic mutations altering drug targets have raised concerns regarding their antibacterial effectiveness [23], 24]. In cases of sepsis, CRKP’s ability to withstand high-dose antibiotic exposure while simultaneously horizontally transferring resistance genes and delivering virulence factors, thereby evading immune surveillance and triggering severe systemic inflammatory responses, is largely attributed to its secreted OMVs [3], 25]. OMVs serve as potent natural markers of bacterial activity; they capture biomolecules from the environment while concurrently transmitting encapsulated enzymes and drug resistance genes [26]. OMVs can easily traverse the blood-brain barrier due to their nanoscale size, enabling long-distance communication independent of the bacterial cell and facilitating the accumulation of specific organ virulence factors [27]. Studies have demonstrated that OMVs released by enterotoxigenic *Escherichia coli* can irreversibly bind to T4 phage, indicating that OMVs can mimic bait to attract attacks intended for the bacterial cell itself [28]. Leveraging the unique pathogenic mechanisms of OMVs, researchers have progressively developed these vesicles as drug carriers or active agents to exert antibacterial effects in recent years. For example, engineered *Pseudomonas aeruginosa* OMVs incorporating β-glycosidase have demonstrated enhanced bactericidal activity against Gram-negative organisms [29]. Consequently, there is an urgent need to investigate the mechanisms underlying CRKP resistance and identify effective targeted resistance proteins. Given this context, focusing on OMVs presents a promising avenue for exploring CRKP resistance mechanisms and discovering effective targeted resistance proteins. With advancements in proteomics, research on OMVs derived from *K. pneumoniae* has gradually deepened [20], 30]. Nevertheless, studies addressing meaningful drug resistance or therapeutic targets through proteomic analysis of OMVs originating from CRKP in sepsis remain exceedingly rare.

In this study, we selected CSKP and CRKP strains isolated from patients with sepsis, and extracted their OMVs for MS analysis to investigate protein differences. The analysis revealed that compared to CSKP, the OMVs secreted by CRKP contained a greater number of proteins, including 140 unique proteins, whereas CSKP-OMVs harbored only 5 unique proteins. Differential expression analysis further revealed a markedly higher number of up-regulated proteins in CRKP-OMVs, indicating substantial remodeling of vesicle cargo associated with carbapenem resistance. These findings emphasize the potential role of OMVs in the adaptive capacity and pathogenicity of CRKP strains [31].

The differential proteins in OMVs were analyzed, and some promising proteins were screened out in combination with historical studies. AdhE mainly displays its functions in fermentation and metabolism, but a large number of studies have confirmed that it has become an important medium for many pathogens to spread virulence factors in direct or indirect ways [32]. When *E. coli* and *Listeria monocytogenes* lack AdhE gene, their host cell adhesion function is severely hit, and the bacterial motility is also weakened [33]. NDK, It is homologous to human NME gene, and its epidemic spread in a variety of pathogens has become an obstacle to the effectiveness of drugs [34]. NME/NDK proteins mainly regulate host immune functions, such as regulating macrophages in an ATP independent manner to change the internal and external environment of host cells [34]. Many studies have shown that NDK is the future star of developing new targeted drugs. TreA has the function of capacity storage, which widely exists in prokaryotes and eukaryotes. Some experiments showed that eliminating the mutation of treA could reduce the virulence of fungi [35]; In addition, knockdown of the treA mutant in extraintestinal *E. coli* strain BEN2908 reduced type 1 pilus formation and bladder colonization [36]. However, there are few studies on *K. pneumoniae*, especially CRKP. AckA is very helpful to the biofilm formation of MRSA, and then strengthens the survival and multiple organ dissemination level of MRSA [37]. After constructing the ackA deletion mutant, the researchers found that the glucose and ATP uptake of the strain was severely destroyed, resulting in the growth atrophy of the strain [38]. At the same time, for *K. pneumoniae*, the PTA-ackA pathway significantly regulates its virulence by regulating global protein acetylation, serum resistance and type 3 pili expression, which has become the direction of many drug target development projects [39]. In our data, the expression of adhE, NDK, treA, ackA in CRKP secreted OMVs was significantly increased, indicating that they also played an important role in bacterial resistance. Among the 140 unique proteins, galE and Ter family seem to be worthy of further study. The data showed that in ESBL-producing *K. pneumoniae*, galE protein expression was greatly increased, and it was an inducer of bacterial virulence enhancement and rapid growth due to its participation in galactose metabolism and LPS production. Therefore, targeted knockdown of galE may be able to destroy the growth of ESBL-producing *K. pneumoniae* [40]*.* Tellurite resistance gene operons (ter) are found in many pathogenic bacteria. Interestingly, it is not a single ter protein that is responsible for tellurite resistance, but the combination of ter members with each other is required for pathogenic activities such as promoting CRKP colonization and high fitness in the gut [41], 42].

In this study, the differential protein functions are mostly related to enzymes, such as Superoxide dismutase and Peroxidase activity, which can be used as experimental indicators to determine the growth resistance trend of *K. pneumoniae* [43], 44]*.* In the main pathways involved in differential proteins, β-alanine is a component of pantothenic acid salts, which participate in the synthesis of CoA [45]. Meaningfully, the pantothenic acid pathway gene panD is missing in CRKP-OMVs. Arginine biosynthesis is a necessary biological process for bacterial growth. The latest data confirms that knocking out the Arg regulator in highly virulent *K. pneumoniae* leads to a decrease in bacterial mucus and an increase in capsule polysaccharide chain length [46]. Among the upregulated pathways involved in CRKP, O-antigen nucleoside sugar biosynthesis and Lipopolysaccharide biosynthesis have the most significant significance. In this study, glaE was significantly elevated in CRKP, and its activity directly affected the supply of UDP galactose, thereby hindering O-antigen synthesis [47]. A large number of phage resistance studies have focused on Lipopolysaccharide O-antigen. One study showed that the necessary reversible receptor of phage exists in Lipopolysaccharide O-antigen, and modifying its wecA and wecG can significantly destroy the adsorption ability and infection efficiency of phage [48].

Due to the presence of numerous pathogen-related molecular patterns and their ability to stimulate immune responses, extensive research has been conducted on modifying OMVs for use as vaccine carriers and immune adjuvants. Among these efforts, the technology for modifying *E. coli* OMVs is relatively advanced. Researchers have successfully engineered wild-type *E. coli* with genes encoding melanin production, resulting in OMVs that significantly enhance anti-tumor effects [49]. Other research teams have utilized *Salmonella*-derived OMVs as carriers, engineering them into antigen-delivery vectors aimed at controlling *Helicobacter pylori* infections [50]. Previous studies focusing on multidrug-resistant *K. pneumoniae* predominantly employed standard strains. however, findings from various lipidomics, proteomics, and RNA sequencing investigations indicate that the function and composition of Gram-negative bacterial vesicles are influenced by both the metabolic state of the bacteria and the surrounding disease environment [9], 51]. Consequently, this study utilizes clinical strains isolated from sepsis patients to examine protein alterations in OMVs following carbapenem resistance development in *K. pneumoniae*.

Although this study provides an in-depth comparative proteomic analysis of OMVs derived from clinical CRKP and CSKP bloodstream isolates, several limitations should be acknowledged. First, only three CRKP and three CSKP strains were included. While PCA and RSD analyses demonstrated good intra-group reproducibility, this small cohort cannot fully capture the genetic and geographic diversity of *K. pneumoniae*, thereby limiting the generalizability of the findings. Second, the study is based solely on discovery proteomics. Although several resistance-associated candidates were enriched in CRKP-OMVs, no functional validation experiments (e.g., gene knockout, enzymatic activity assays, or host–cell interaction studies) were conducted to establish causal roles in resistance or virulence. Thus, the results should be interpreted as hypothesis-generating.

Despite these limitations, the present findings provide a valuable foundation for future mechanistic studies. Subsequent work will aim to validate the functional relevance of key candidate proteins, delineate their contribution to CRKP pathogenicity and stress adaptation, and determine whether targeted perturbation can alter OMV composition or bacterial phenotype. In addition, the differential proteins identified here may enable engineering of *K. pneumoniae*–derived OMVs for potential applications such as antigen delivery, vaccine development, or therapeutic modulation of antibiotic-resistant strains in sepsis. Together, these directions may help translate OMV-based biological insights into clinically meaningful strategies against multidrug-resistant *K. pneumoniae*.
